# The most influential articles on stem cells in intervertebral disc degeneration

**DOI:** 10.1186/s12891-024-07253-z

**Published:** 2024-02-08

**Authors:** Shuxi Ye, Rongchun Chen, Jiangyou Shi, Yaohong Wu

**Affiliations:** Department of Spine Surgery, Ganzhou People’s Hospital, No 16, Meiguan Road, Ganzhou, 341099 China

**Keywords:** Stem cells, Intervertebral disc degeneration, Publication, Web of science, Citation

## Abstract

**Background:**

Stem cell-related studies have been increasingly conducted to facilitate the regeneration of degenerative discs. However, analyses of high-impact articles focused on this topic are rare. This study aimed to determine and summarize the most-cited studies examining stem cells in the context of intervertebral disc degeneration (IDD).

**Methods:**

We searched the Web of Science (WoS) database for stem cell-related articles in IDD, and the 50 highest-cited papers were summarized. A correlation analysis was conducted to determine the relationship among WoS citations, Altmetric Attention Score (AAS), and Dimensions.

**Results:**

The number of citations of the top 50 manuscripts ranged from 92 to 370. The top three countries were the United States (14), China (10), and Japan (9). *Spine* (12) was the most prevalent journal, and this was followed by *Biomaterials* (6). Bone marrow-derived stem cells were the most common subject (38), and they were followed by nucleus pulposus-derived stem cells (4) and annulus fibrosus-derived stem cells (4). Humans were the most studied species (31), and the next most studied were rabbits (9) and rats (7). There was a very high correlation between WoS and Dimension citations (*p* < 0.001, *r* = 0.937).

**Conclusions:**

For the first time, the highest impact articles examining stem cells in the context of IDD were assessed together. The current study provides a deepened understanding of historical studies focused on stem cells in IDD and is beneficial for future studies in this field.

## Introduction

Low back pain (LBP) affects up to 84% of adults during their lifetime and is believed to be the most common musculoskeletal disorder that causes hospital visits [1–3]. LBP is the predominant cause of sick leave and subsequent disability worldwide, thus imposing an immense socioeconomic burden [4, 5]. The total annual cost of LBP in the United States is estimated to exceed $100 billion [6]. Intervertebral disc degeneration (IDD) is a predominant cause of LBP. Contemporary treatments for IDD aimed at alleviating symptoms or minimizing disability often do not offer satisfactory outcomes for a large number of patients [5–7]. Neither surgical nor non-surgical interventions can hinder the progress of IDD or reverse it to regain functional discs [3, 7, 8]. Hence, new treatment strategies that focus on curing IDD are required.

Stem cell biology and its applications in IDD have received increasing attention due to the limitations of current invention options [7–13]. With the rapid development in stem cell research, a substantial number of studies have been conducted on IDD [9–11, 13]. Several important studies exhibit great potential for promoting stem cell research in the context of IDD [8–11]. The tendencies of a certain field are commonly reflected in high impact studies [14–18]. The evaluation of these studies can help researchers and clinicians to rapidly identify the most influential papers in a specific field and deepen their research or identify novel directions based on these classic studies [17–20]. Analyses of the most frequently cited papers have been conducted in various fields [14–26]. Nevertheless, such investigations have not been applied to stem cell research in IDD. The purpose of the present study was to determine the 50 most frequently cited documents on stem cells in IDD and to investigate their features.

## Methods

### Search strategy

Approval from the Institutional Review Board was not required, as no studies were conducted using humans or animals. The Web of Science (WoS) database was used as the literature source. On April 5, 2023, this database was searched using the terms “stem cell”, “stromal cell”, “progenitor cell”, “precursor cell”, “intervertebral disc”, “intervertebral disk”, “annulus fibrosus”, “nucleus pulposus”, and “endplate”. The search was not limited by publication date, article type, or language. The identified papers were listed in descending order based on WoS citations. Articles investigating the effects of stem cells on disc degeneration and regeneration, including stem cell transplantation, resident stem cells, and stem cell-derived exosomes, were included. Two authors independently screened the papers for relevance to stem cells in the context of IDD. In cases of disagreements regarding study selection between the two authors, a third author made the final decision. The 50 highest-cited articles on stem cells in IDD were included.

### Data management

After the final top list was determined, data extraction and analysis were performed independently by two authors. If a consensus was not achieved, a third author was consulted to make the final decision. The extracted data included title, year, citation count, journal, article type, country, institution, author, source species, and stem cells. Dimension citations and the Altmetric Attention Score (AAS) were identified using the Dimensions database (www.dimensions.ai).

### Statistical analysis

Descriptive statistics, including total counts, average counts, and percentages, were used to analyze the extracted data. A correlation analysis was performed to detect the relationship among WoS citations, AAS, and Dimension citations. A correlation coefficient of Pearson’s test (r) < 0.3 was defined as poor, 0.3–0.5 was defined as low, 0.5–0.7 was defined as moderate, 0.7–0.9 was defined as high, and > 0.9 was defined as very high. *P* < 0.05 was indicative of statistical significance.

## Results

### The top 50 list

The 50 most-cited papers on stem cells in IDD are listed in Table 1. The number of WoS citations per paper ranged from 92 to 370 (mean of 170). The most cited study was reported in *Biomaterials* in 2003, and it was also the oldest study. The most recent manuscript (ranked 38th with 111 citations) was published in *Theranostics*. The number of dimension citations ranged from 73 to 340 (mean of 164). The highest AAS was 19 (mean of 5). Eight studies (16%) had no AAS. All studies were published in English.

**Table 1:** The top 50 works on stem cells in the intervertebral disc degeneration

Rank	First Author	Year	Article	Journal	WoS Citations	Dimensions	AAS
1	Sakai D	2003	Transplantation of mesenchymal stem cells embedded in Atelocollagen((R)) gel to the intervertebral disc: a potential therapeutic model for disc degeneration	*Biomaterials*	370	335	6
2	Sakai D	2006	Regenerative effects of transplanting mesenchymal stem cells embedded in atelocollagen to the degenerated intervertebral disc	*Biomaterials*	324	304	6
3	Orozco L	2011	Intervertebral disc repair by autologous mesenchymal bone marrow cells: a pilot study	*Transplantation*	320	340	13
4	Sakai D	2005	Differentiation of mesenchymal stem cells transplanted to a rabbit degenerative disc model - Potential and limitations for stem cell therapy in disc regeneration	*Spine*	312	293	19
5	Risbud MV	2004	Differentiation of mesenchymal stem cells towards a nucleus pulposus-like phenotype in vitro: implications for cell-based transplantation therapy	*Spine*	298	262	3
6	Sakai D	2012	Exhaustion of nucleus pulposus progenitor cells with ageing and degeneration of the intervertebral disc	*Nature Communications*	277	281	8
7	Richardson SM	2006	Intervertebral disc cell-mediated mesenchymal stem cell differentiation	*Stem Cells*	275	242	3
8	Crevensten G	2004	Intervertebral disc cell therapy for regeneration: mesenchymal stem cell implantation in rat intervertebral discs	*Annals of Biomedical Engineering*	265	264	6
9	Risbud MV	2007	Evidence for skeletal progenitor cells in the degenerate human intervertebral disc	*Spine*	248	225	6
10	Sakai D	2015	Stem cell therapy for intervertebral disc regeneration: obstacles and solutions	*Nature Reviews Rheumatology*	245	265	18
11	Steck E	2005	Induction of intervertebral disc-like cells from adult mesenchymal stem cells	*Stem Cells*	242	217	6
12	Richardson SM	2008	Human mesenchymal stem cell differentiation to NP-like cells in chitosan-glycerophosphate hydrogels	*Biomaterials*	217	189	0
13	Dang JM	2006	Temperature-responsive hydroxybutyl chitosan for the culture of mesenchymal stem cells and intervertebral disk cells	*Biomaterials*	208	193	0
14	Hiyama A	2008	Transplantation of mesenchymal stem cells in a canine disc degeneration model	*Journal of Orthopaedic Research*	206	202	6
15	Vadala G	2012	Mesenchymal stem cells injection in degenerated intervertebral disc: cell leakage may induce osteophyte formation	*Journal of Tissue Engineering and Regenerative Medicine*	196	206	7
16	Yoshikawa T	2010	Disc regeneration therapy using marrow mesenchymal cell transplantation: a report of two case studies	*Spine*	180	186	0
17	Sobajima S	2008	Feasibility of a stem cell therapy for intervertebral disc degeneration	*Spine Journal*	172	165	5
18	Blanco JF	2010	Isolation and characterization of mesenchymal stromal cells from human degenerated nucleus pulposus comparison with bone marrow mesenchymal stromal cells from the same subjects	*Spine*	170	160	3
19	Minogue BM	2010	Characterization of the human nucleus pulposus cell phenotype and evaluation of novel marker gene expression to define adult stem cell differentiation	*Arthritis and Rheumatism*	166	170	3
20	Henriksson HB	2009	Identification of cell proliferation zones, progenitor cells and a potential stem cell niche in the intervertebral disc region: a study in four species	*Spine*	161	160	6
21	Richardson SM	2006	The differentiation of bone marrow mesenchymal stem cells into chondrocyte-like cells on poly-L-lactic acid (PLLA) scaffolds	*Biomaterials*	160	142	3
22	Henriksson HB	2009	Transplantation of human mesenchymal stems cells into intervertebral discs in a xenogeneic porcine model	*Spine*	159	161	3
23	Nesti LJ	2008	Intervertebral disc tissue engineering using a novel hyaluronic acid-nanofibrous scaffold (HANFS) amalgam	*Tissue Engineering Part A*	154	165	3
24	Ganey T	2009	Intervertebral disc repair using adipose tissue-derived stem and regenerative cells experiments in a canine model	*Spine*	150	149	5
25	Leung VYL	2006	Regeneration of intervertebral disc by mesenchymal stem cells: potentials, limitations, and future direction	*European Spine Journal*	147	146	3
26	Cheng XF	2018	Mesenchymal stem cells deliver exogenous miR-21 via exosomes to inhibit nucleus pulposus cell apoptosis and reduce intervertebral disc degeneration	*Journal of Cellular and Molecular Medicine*	146	156	3
27	Liu LT	2011	Characteristics of stem cells derived from the degenerated human intervertebral disc cartilage endplate	*PLoS One*	143	98	6
28	Strassburg S	2010	Co-culture induces mesenchymal stem cell differentiation and modulation of the degenerate human nucleus pulposus cell phenotype	*Regenerative Medicine*	134	113	6
29	Stoyanov JV	2011	Role of hypoxia and growth and differentiation factor-5 on differentiation of human mesenchymal stem cells towards intervertebral nucleus pulposus-like cells	*European Cells & Materials*	133	121	0
30	Zhang YG	2005	Bone mesenchymal stem cells transplanted into rabbit intervertebral discs can increase proteoglycans	*Clinical Orthopaedics and Related Research*	133	138	3
31	Serigano K	2010	Effect of cell number on mesenchymal stem cell transplantation in a canine disc degeneration model	*Journal of Orthopaedic Research*	131	131	0
32	Liu C	2015	The effect of the fibre orientation of electrospun scaffolds on the matrix production of rabbit annulus fibrosus-derived stem cells	*Bone Research*	129	73	0
33	Wuertz K	2008	Behavior of mesenchymal stem cells in the chemical microenvironment of the intervertebral disc	*Spine*	128	120	3
34	Huang YC	2013	The effects of microenvironment in mesenchymal stem cell-based regeneration of intervertebral disc	*Spine Journal*	125	123	3
35	Yang F	2009	Mesenchymal stem cells arrest intervertebral disc degeneration through chondrocytic differentiation and stimulation of endogenous cells	*Molecular Therapy*	118	118	3
36	Lu K	2017	Exosomes as potential alternatives to stem cell therapy for intervertebral disc degeneration: in-vitro study on exosomes in interaction of nucleus pulposus cells and bone marrow mesenchymal stem cells	*Stem Cell Research & Therapy*	117	122	4
37	Noriega DC	2017	Intervertebral disc repair by allogeneic mesenchymal bone marrow cells: a randomized controlled trial	*Transplantation*	117	124	4
38	Liao ZW	2019	Exosomes from mesenchymal stem cells modulate endoplasmic reticulum stress to protect against nucleus pulposus cell death and ameliorate intervertebral disc degeneration in vivo	*Theranostics*	111	118	0
39	Nerurkar NL	2010	Engineered disc-like angle-ply structures for intervertebral disc replacement	*Spine*	106	118	13
40	Vadala G	2008	Coculture of bone marrow mesenchymal stem cells and nucleus pulposus cells modulate gene expression profile without cell fusion	*Spine*	106	108	3
41	Calderon L	2010	Type II collagen-hyaluronan hydrogel - a step towards a scaffold for intervertebral disc tissue engineering	*European Cells & Materials*	104	104	9
42	Miyamoto T	2010	Intradiscal transplantation of synovial mesenchymal stem cells prevents intervertebral disc degeneration through suppression of matrix metalloproteinase-related genes in nucleus pulposus cells in rabbits	*Arthritis Research & Therapy*	104	108	3
43	Korecki CL	2010	Notochordal cell conditioned medium stimulates mesenchymal stem cell differentiation toward a young nucleus pulposus phenotype	*Stem Cell Research & Therapy*	103	100	3
44	Shen BJ	2009	BMP-2 enhances TGF-beta 3-mediated chondrogenic differentiation of human bone marrow multipotent mesenchymal stromal cells in alginate bead culture	*Tissue Engineering Part A*	103	93	6
45	Acosta FL	2011	Porcine intervertebral disc repair using allogeneic juvenile articular chondrocytes or mesenchymal stem cells	*Tissue Engineering Part A*	100	113	4
46	Pettine KA	2015	Percutaneous injection of autologous bone marrow concentrate cells significantly reduces lumbar discogenic pain through 12 months	*Stem Cells*	99	118	6
47	Feng G	2010	Multipotential differentiation of human anulus fibrosus cells: an in vitro study	*Journal of Bone and Joint Surgery-American Volume*	98	89	3
48	Yang SH	2008	In vitro study on interaction between human nucleus pulposus cells and mesenchymal stem cells through paracrine stimulation	*Spine*	95	78	0
49	Clarke LE	2014	Growth differentiation factor 6 and transforming growth factor-beta differentially mediate mesenchymal stem cell differentiation, composition, and micromechanical properties of nucleus pulposus constructs	*Arthritis Research & Therapy*	94	99	1
50	Frith JE	2013	An injectable hydrogel incorporating mesenchymal precursor cells and pentosan polysulphate for intervertebral disc regeneration	*Biomaterials*	92	101	15

*WoS, Web of Science; AAS, Altmetric Attention Score*

### Year of publication

**Fig. 1:**
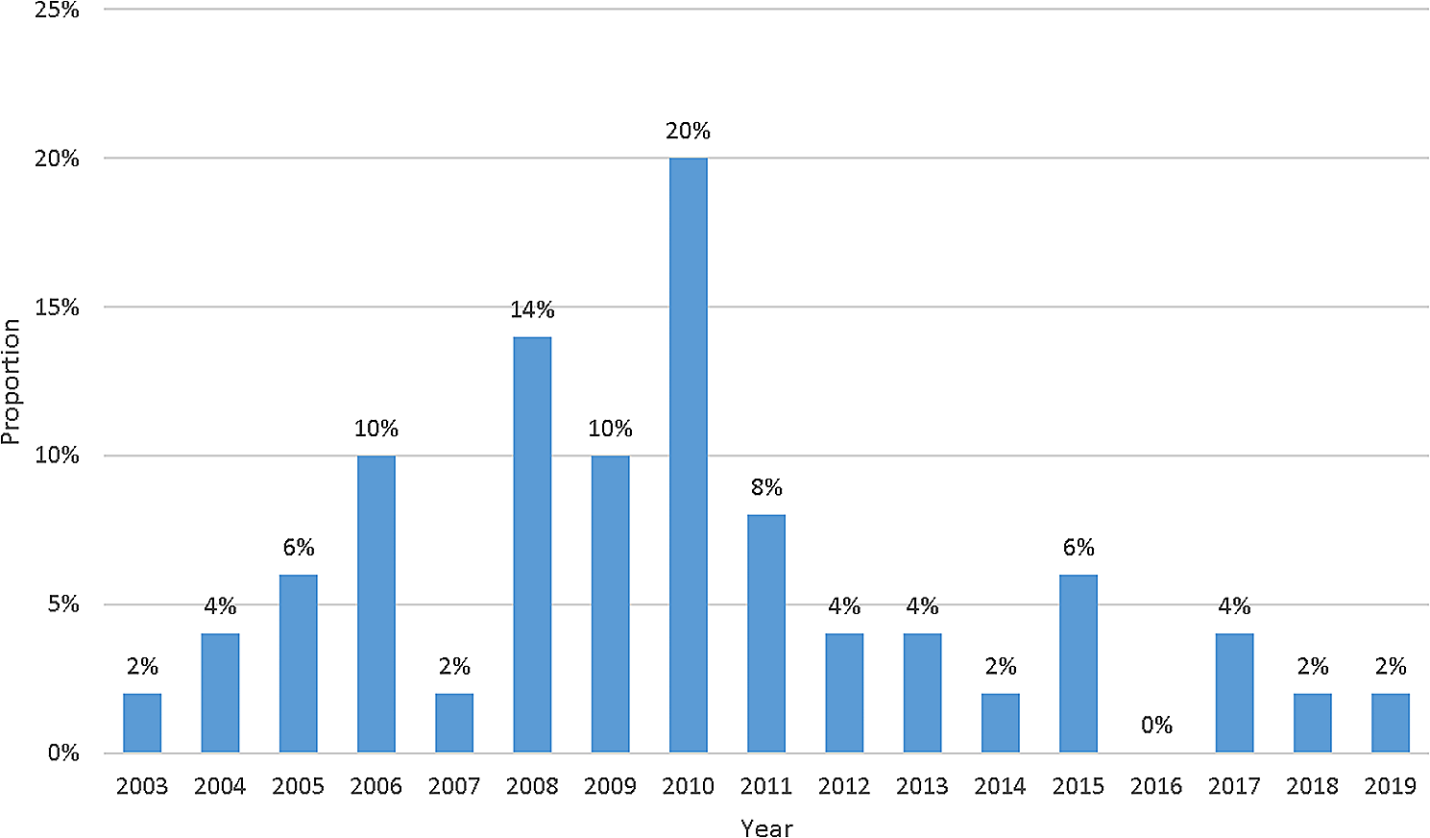
The proportion of the annual number of top 50 studies on stem cells in the intervertebral disc degeneration

The proportion of the annual number of the top 50 studies is presented in Fig. 1. The top 50 studies were published between 2003 and 2019. The most prolific year was 2010 with 10 papers (20%), and this was followed by 2008 with seven papers (14%). The number of influential stem cell-related studies was the most prominent from 2008 to 2011 (26, 52%). Among the top 50 papers, none were published in 2016.

### Article type

**Fig. 2:**
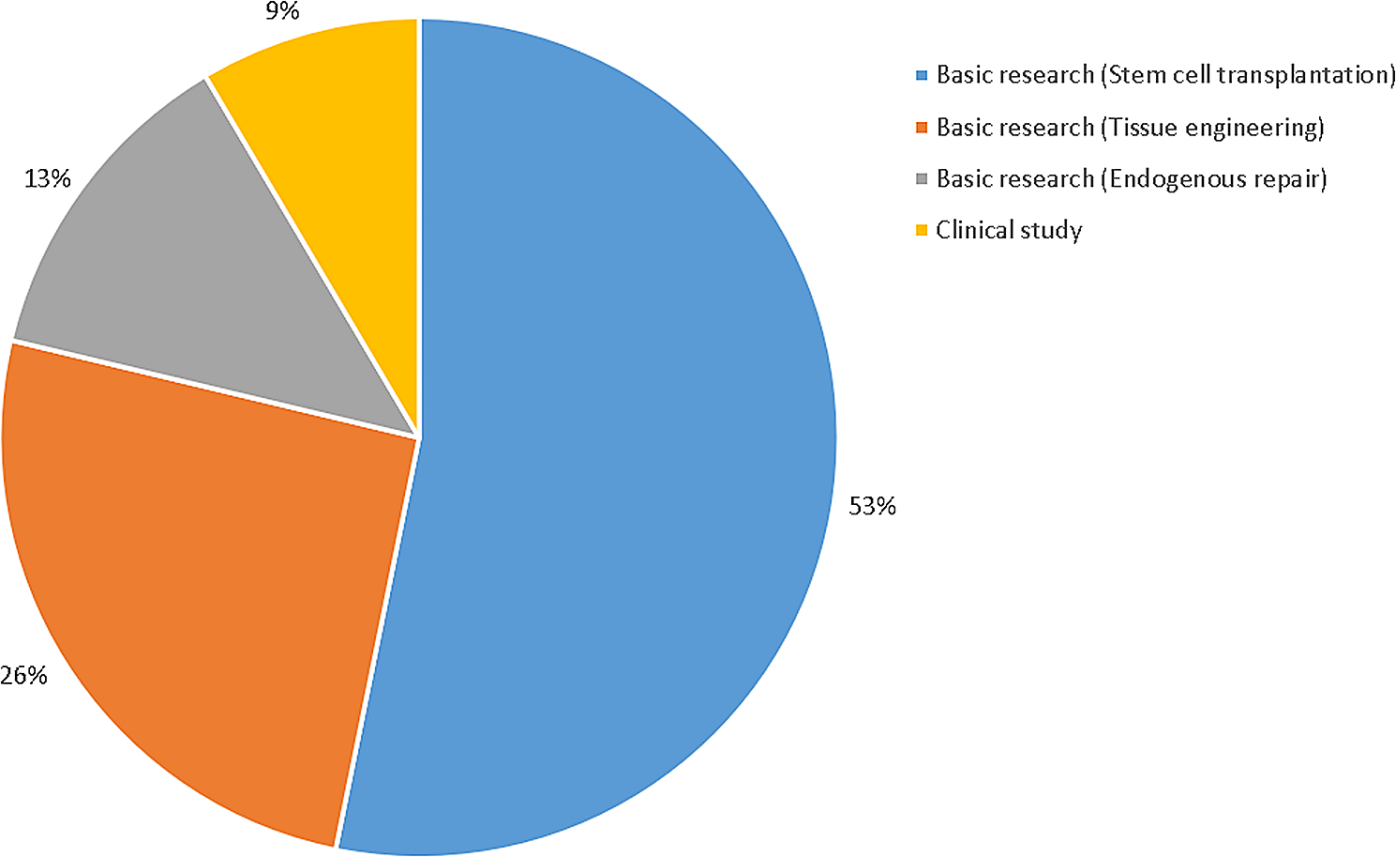
The topics of the original research of the top 50 studies

Three papers (6%) were reviewed, and the remaining 47 (94%) were original research that included clinical studies (4, 9%) and basic research (43, 91%). The most discussed topic in the basic research was stem cell transplantation (25, 53%), and this was followed by tissue engineering (12, 26%) and endogenous repair (6, 13%). The topics of the original study are presented in Fig. 2.

### Source journal

Table 2 lists the journals in which the top 50 articles were published. A total of 24 journals produced 50 articles. Ten journals published at least two studies. The most prevalent journal was *Spine* with 12 papers, and this was followed by *Biomaterials* (6), *Stem cells* (3), and *Tissue Engineering Part A* (3). Moreover, *Spine* possessed the highest total citations (2113), and this was followed by *Biomaterials* (1371). Of the 24 journals, *Nature Reviews Rheumatology* possessed the highest impact factor (20.543), and this was followed by *Nature Communications* (14.919) and *Bone Research* (13.567).

**Table 2:** Journal of origin

Journal title	No. of papers	Total citations
Spine	12	2113
Biomaterials	6	1371
Stem Cells	3	616
Tissue Engineering Part A	3	357
Stem Cell Research & Therapy	2	220
Arthritis Research & Therapy	2	198
Transplantation	2	437
Spine Journal	2	297
European Cells & Materials	2	237
Journal of Orthopaedic Research	2	337
Nature Reviews Rheumatology	1	245
Nature Communications	1	277
Bone Research	1	129
Theranostics	1	111
Molecular Therapy	1	118
Journal of Cellular and Molecular Medicine	1	146
Journal of Bone and Joint Surgery-American Volume	1	98
Clinical Orthopaedics and Related Research	1	133
Journal of Tissue Engineering and Regenerative Medicine	1	196
Annals of Biomedical Engineering	1	265
Regenerative Medicine	1	134
PLoS ONE	1	143
European Spine Journal	1	147
Arthritis and Rheumatism	1	166

### Country distribution

Table 3 lists the countries of the top 50 studies. Eleven countries produced the 50 most-cited papers. The United States ranked first with 14 papers, and this was followed by China (10) and Japan (9). The top three countries in terms of total citations were the United States (2,235), Japan (2,149), and China (1,264). Seven countries published at least two papers each. Among these countries, Japan possessed the highest number of citations per paper (238.78), and this was followed by Spain (202.33) and the United Kingdom (174.33).

**Table 3:** Countries of the top 50 works

Countries	No. of papers	Total citations
United States	14	2235
China	10	1264
Japan	9	2149
United Kingdom	6	1046
Spain	3	607
Sweden	2	320
Australia	2	195
Germany	1	242
Italy	1	196
Switzerland	1	133
Ireland	1	104

### Institution of origin

The affiliated institutions that contributed two or more papers are listed in Table 4. There were ten institutions on the list. Tokai University School of Medicine with seven papers possessed the leading publication record, and this was followed by The University of Manchester (6) and The University of Hong Kong (3). The Tokai University School of Medicine also possessed the highest total citations (1,865), and this was followed by The University of Manchester (1,046) and Thomas Jefferson University (546). Regarding average citations, Thomas Jefferson University ranked first (273.00), and this was followed by Tokai University School of Medicine (266.43) and the University of Valladolid and CSIC (218.50).

**Table 4:** Institutions with two or more papers among the top 50 works

Institutions	No. of papers	Total citations	Average citations
Tokai University School of Medicine	7	1865	266.43
The University of Manchester	6	1046	174.33
The University of Hong Kong	3	390	130.00
Thomas Jefferson University	2	546	273.00
University of Valladolid and CSIC	2	437	218.50
University of California	2	365	182.50
University of Gothenburg	2	320	160.00
University of Pittsburgh School of Medicine	2	278	139.00
Third Military Medical University	2	260	130.00
University of Vermont	2	231	115.50

### Corresponding author

Table 5 lists the corresponding authors of two or more papers. Sakai authored seven papers and topped the list, and this was followed by Hoyland JA (6). Sakai D also possessed the highest number of citations (1,865), and this was followed by Hoyland JA (1,046). With respect to average citations, Risbud MV was the leader (273.00), and this was followed by Sakai D (266.43) and Garcia-Sancho J (218.50).

**Table 5:** Authors with two or more papers of the top 50 works

Corresponding authors	No. of papers	Total citations	Average citations
Sakai D	7	1865	266.43
Hoyland JA	6	1046	174.33
Risbud MV	2	546	273.00
Garcia-Sancho J	2	437	218.50
Lotz JC	2	365	182.50
Brisby H	2	320	160.00
Kang JD	2	279	139.00
Cheung KMC	2	265	132.50

### Classification of species

**Fig. 3:**
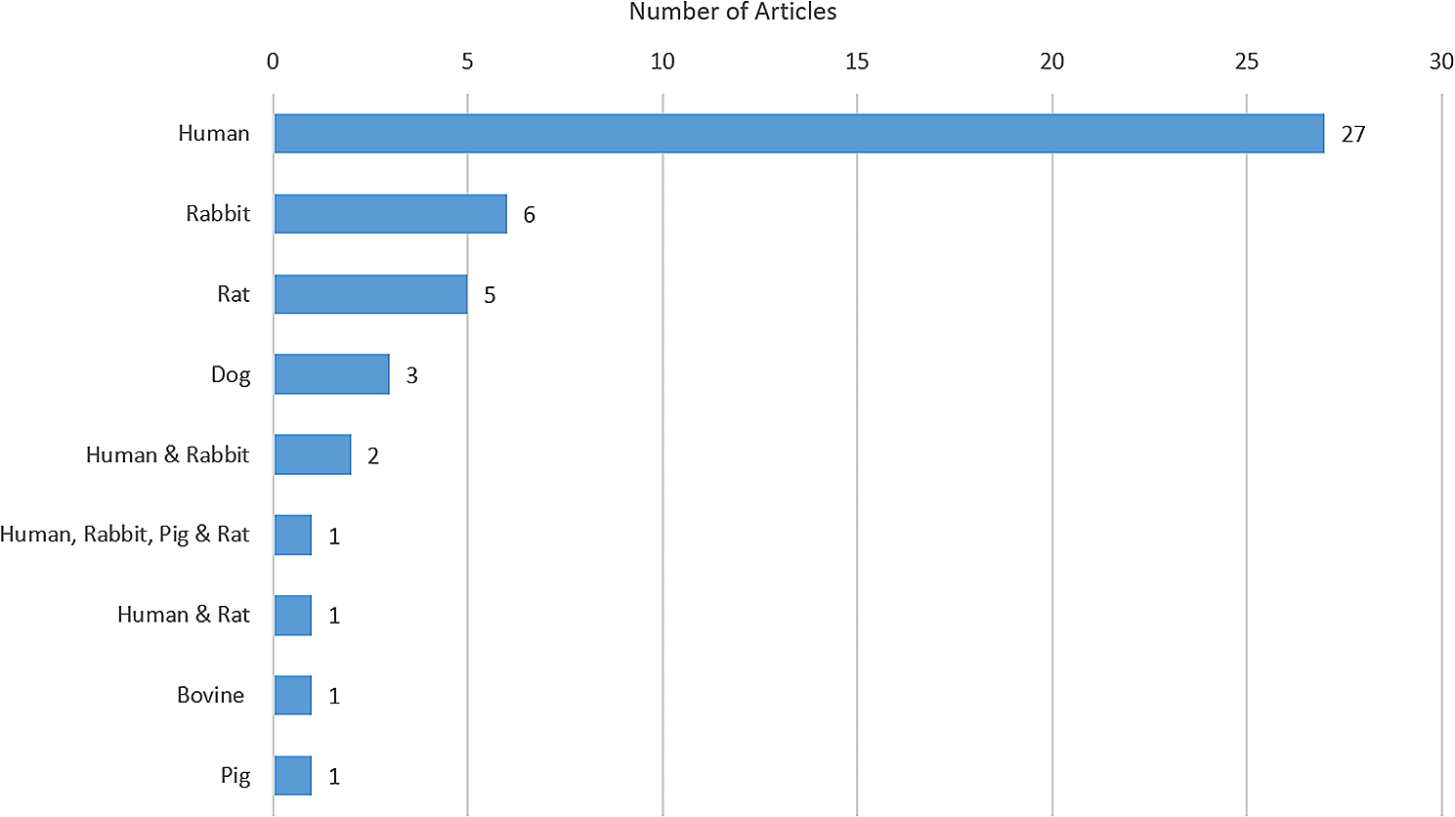
The species investigated in the original research

The classification of stem cell species in the 47 original studies is presented in Fig. 3. Six species were involved in these 47 studies, including humans, rabbits, rats, dogs, pigs, and animals. The majority of studies (43) examined one species, whereas the other four studies investigated two or more species. Humans (31 papers) were the most studied species, and this was followed by rabbits (9) and rats (7).

### Category of stem cells

**Fig. 4:**
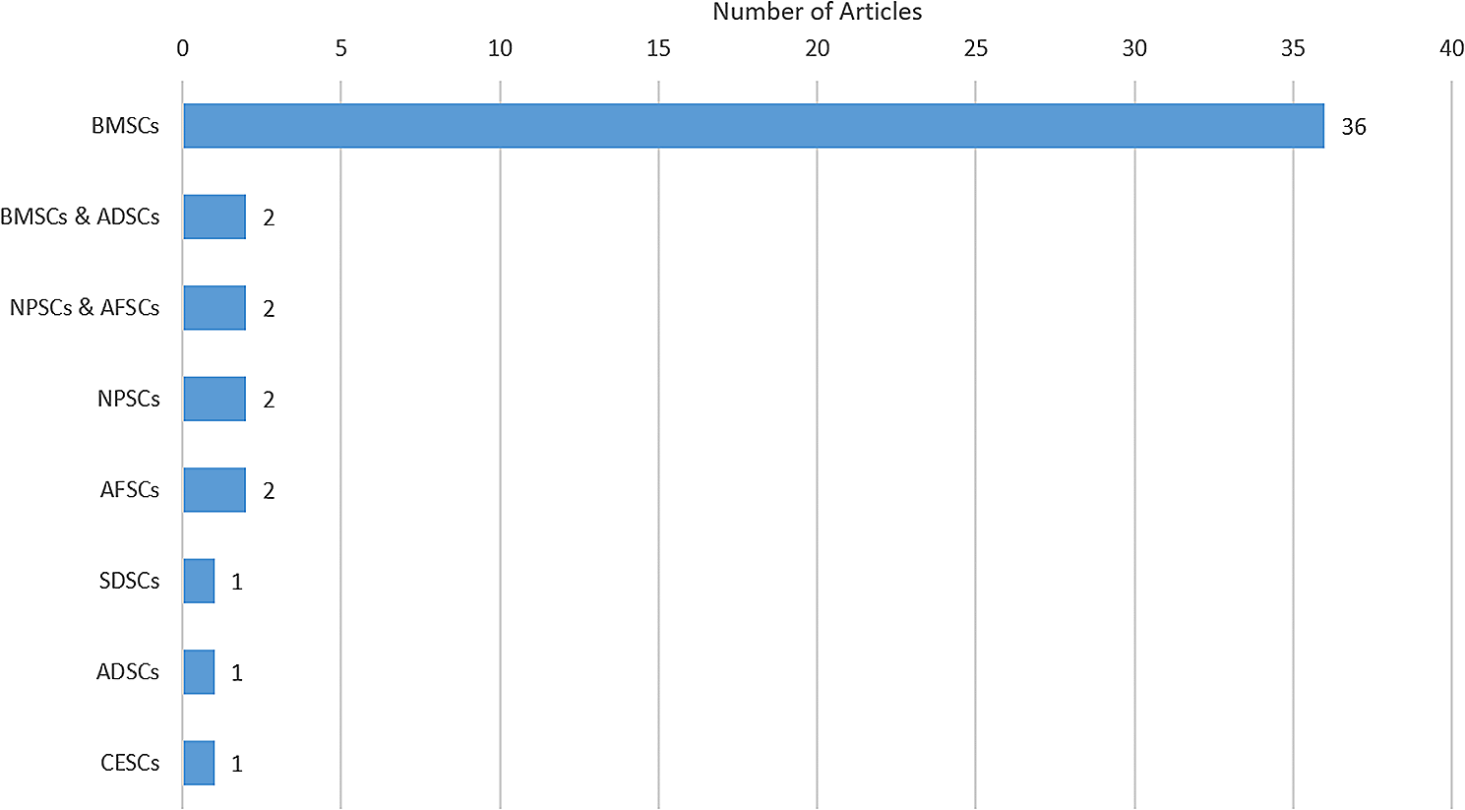
The stem cells discussed in the original research

Figure 4 depicts stem cells discussed in the 47 original studies. Most studies (45) investigated one type of stem cell, while other studies (2) examined two types of stem cells. Six types of stem cells were included in these 47 studies, including bone marrow-derived stem cells (BMSCs), adipose-derived stem cells (ADSCs), nuclear pulposus-derived stem cells (NPSCs), cartilage endplate-derived stem cells (CESCs), annulus fibrosus-derived stem cells (AFSCs), and synovial-derived stem cells (SDSCs). BMSCs (38) were the most discussed stem cells, and this was followed by NPSCs (4) and AFSCs (4).

### Correlation analysis

**Fig. 5:**
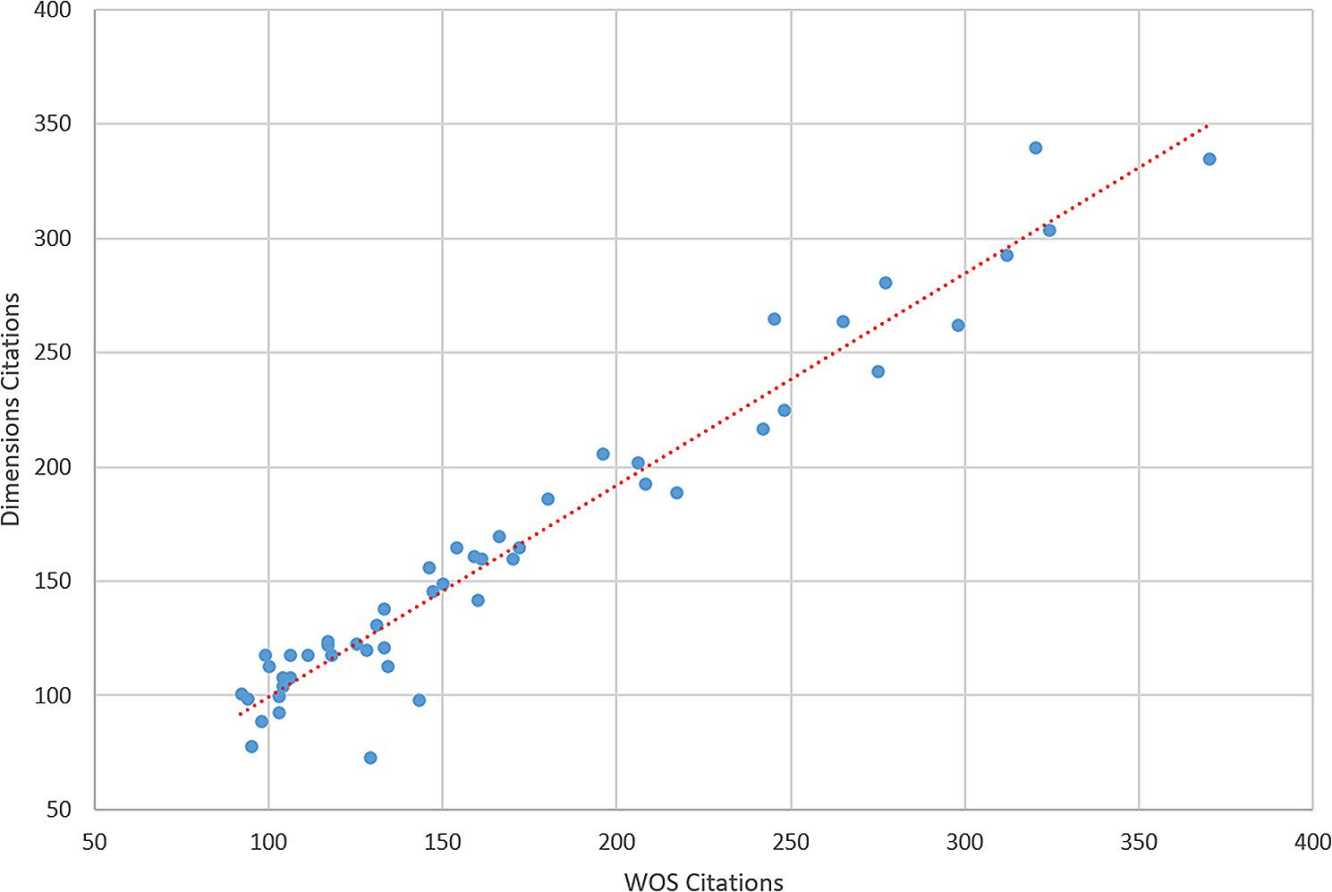
Scatter plot evaluating the correlation between WoS and dimensions citations

The citation counts of the top 50 studies in WoS were highly correlated with their citations in Dimensions (*r* = 0.973, *p* < 0.001). Figure 5 indicates a clear linear correlation between the WoS and dimension citations. Additionally, a low correlation was observed between the number of citations in the WoS and the AAS (*r* = 0.340, *p* = 0.016).

## Discussion

LBP has become the leading cause of disability and severely influences the quality of life of patients while placing a huge burden on the society and economy [2, 3, 5, 6]. LBP is primarily associated with IDD [9–12]. Current strategies for IDD are limited and cannot solve the problem [11, 12]. Therefore, there is a crucial need to develop new treatment options to delay IDD and restore disc functions [7, 8, 11–13]. Progress in stem cell research may provide a potent strategy for IDD treatment [8–10, 12, 13]. Additionally, the most influential works may alter clinical practice and motivate discussions, disputes, and further studies [17–20]. Although the majority of publications analyzing the highest-cited works have been reported in many fields [14–26], there have been no such reports in the context of stem cell research on IDD. To the best of our knowledge, this is the first study to determine and analyze the greatest impact works on stem cells in IDD.

The number of citations of the 50 most cited works typically varies across fields [21–26]. The citation counts of the fifty highest cited papers focused on rotator cuff tears were between 253 and 1,558 [22], and those on orthopedic shoulder surgery were between 192 and 1,211 [24]. Both are much higher than the citations in this study. Possible reasons for these findings are that the number of investigators, the impact of journals, and the number of papers vary across fields [21–26].

We observed that the oldest paper among the top 50 was published in 2003. This result is dissimilar to those observed in other fields [21–26]. For example, the oldest of the 50 most-cited papers on anterior cruciate ligament injury was published in 1941 [23]. This may be due to the observation that the history of scientific research in some fields is longer than it is in others. More than half of the top 50 articles were published between 2008 and 2011. These findings suggest that stem cell research on IDD is a new field of development.

The top 50 articles were published in the English language. This confirms that English is the most important and influential language in the scientific community [17, 21–23, 25, 26]. *Spine* published the largest number of studies, and this was followed by *Biomaterials*, *Stem cells*, and *Tissue Engineering Part A.* This indicates that these journals exerted the greatest influence on stem cell research in IDD. One possibility is that the investigators tend to submit their vital work to high impact journals in their fields [21–26]. Another possibility is that investigators tend to cite papers published in important journals [14–20]. Moreover, the top four popular journals published nearly half of the 50 most-cited papers. This finding indicates that high-impact studies are centered on a small number of important journals [15–17, 25, 26]. Additionally, journals possessing high impact factors such as *Nature Reviews Rheumatology*, *Nature Communications*, and *Bone Research* published at least one paper on stem cells in IDD. This suggests that high-quality studies on this topic can be accepted in high-impact journals [15, 17, 19, 20].

The 50 highest impact studies were published by authors from 11 countries. The top three countries (the United States, China, and Japan) produced 33 papers and accounted for 66% of the top 50 studies. This indicates that high-impact work is concentrated in a few countries. With the exception of China, most of these countries are developed. This indicates that the economic status of countries is associated with the research output of high-impact studies [14, 21, 23, 25]. Therefore, there is a need to improve the quality of works in non-developed countries.

Unsurprisingly, the United States has been the most fruitful country for stem cell research in the context of IDD. The observation that the United States is the most powerful country in terms of scientific productivity has been demonstrated in many fields [14, 18, 19, 22–26]. This finding may be attributed to the many advantages of the United States, including a large number of researchers and sufficient funds [14–23, 25, 26].

Certain institutions and authors possess excellent records among the top 50. Sakai D. at the Tokai University School of Medicine ranked first with the highest total and average citations. It indicates that this author is the most influential author in the field of stem cell research on IDD. Moreover, half of the top ten papers were published by Sakai, and this further emphasizes the high quality of his works.

Humans are the most investigated species in this topic. This indicates that the majority of stem cells were isolated from humans. This may be due to the knowledge that spinal surgery is typically performed on patients with IDD, and the disc tissue is obtained by a discectomy procedure or minimally invasive surgery [1, 5, 9, 11, 13]. These human stem cells may be helpful in the rapid progression of translational medicine for stem cell research focused on IDD. BMSCs are the most widely studied type of stem cell. This may be due to the observation that BMCSs exhibit excellent biological activities and are easy to obtain with minimal injury [3, 12]. However, with a deeper understanding of IDD, increasing reports have demonstrated that the harsh microenvironment of the degenerated disc inhibits the application of BMSCs [10, 12]. Endogenous repair using resident stem cells such as NPSCs, AFSCs, and CESCs has attracted increasing attention due to their better tolerance to disc conditions [9, 10]. Research examining resident stem cells in IDD is predicted to grow rapidly in the near future.

Traditional indicators of academic influence, including impact factors and citations, provide an important view of studies [21, 22, 27]. Nevertheless, social media substantially alters knowledge sharing [15, 16, 27, 28]. Worldwide platforms such as Twitter and Facebook allow investigators to share their works with many more readers that may not be restricted to the academic field and may not be reflected in traditional indicators [27, 28]. In this study, the AAS of 16% of the included studies was zero, and this indicates that these studies had no online activities. Moreover, the citation counts of the top 50 studies in WoS were highly correlated with their citations in the Dimensions (*r* = 0.973, *p* < 0.001). This result is similar to those of previous publications in other fields [15, 16]. This suggests that this new database could provide an alternative to WoS and could compensate for the bias of Altmetric due to rapid changes in social media. Altmetrics can be used as a useful index to investigate the impact of scientific work on society but not as a reliable index of the quality of work [15, 16, 27, 28]. A low correlation was demonstrated between the number of citations in the WoS and AAS (*r* = 0.340, *p* = 0.016), and this is inconsistent with the findings of similar publications [15, 16]. The correlations in these publications have been reported to be poor. This may indicate that the correlation between WoS and AAS citation counts varies in different fields. Moreover, this may be attributed to the observation that different databases cover different journals, and this may affect the citation counts of articles [27]. Therefore, different databases can be used to assess different aspects of the studies.

In recent decades, considerable efforts have been directed toward basic research aimed at regenerating the intervertebral disc [11, 12]. Our study determined that stem cell transplantation, tissue engineering, and endogenous repair are the main regeneration strategies used in basic research. Stem cell transplantation has emerged as an attractive alternative to conventional conservative, surgical, and pharmacological approaches for treating IDD [13]. The rationale behind intradiscal stem cell transplantation is twofold. First, it aims to augment the cellularity of the nucleus pulposus by facilitating the differentiation of transplanted stem cells into functional nucleopulpocytes, and second, it aims to bolster the activity of existing nucleopulpocytes via supportive secretory functions [29]. The introduction of apt stem cells possesses the potential to restore and produce disc tissues with characteristics similar to those of the original [30]. Another promising approach that has been extensively explored is tissue engineering [29]. The use of cellular scaffolds is a crucial factor dictating the success or failure of IDD regeneration [30]. The hostile environment of the degenerated disc critically influences stem cell survival, metabolism, and differentiation, thus potentially curtailing or nullifying stem cell regenerative capabilities [29]. To overcome such challenges, tissue engineering endeavors to replicate the natural microenvironment of the disc by combining biomaterials, soluble factors, and functional cells to recreate the biological and biomechanical properties of the native intervertebral disc [29, 30]. Recently, the discovery and identification of resident stem cells in intervertebral discs has resulted in increased interest in endogenous repair strategies [31, 32]. Resident stem cells are considered to be a promising source for tissue regeneration and offer the advantage of potentially surmounting hurdles associated with exogenous cell therapies [9]. A simple and effective strategy involves mitigating the apoptosis and senescence of indigenous stem cells within the disc that may be induced by various factors during IDD or by directly enhancing the vitality and differentiation capacity of these stem cells [32]. Another strategy for endogenous repair may involve the direct replenishment of the native stem cell population [31, 32]. Nevertheless, endogenous repair remains a topic of preclinical studies and requires further investigation.

Our study identified the four most influential clinical studies that have used stem cell therapy to treat IDD. Autologous BMSCs were used for the first time to treat IDD in two patients experiencing back and leg pain [3, 33]. This treatment involves transplantation of a collagen sponge laden with BMSCs into a degenerated disc. Two years after the transplantation, both patients experienced diminished pain and elevated intradiscal water content. However, no enhancement in disk height was observed [33]. Despite the small sample size, lack of a control group, and short follow-up period, this study demonstrates for the first time that BMSCs intradiscal transplantation is a safe procedure with considerable potential for IDD treatment [3]. Moreover, a pilot study involving 10 patients with LBP refractory to conservative management was conducted [34]. These patients received injections of autologously expanded BMSCs into the nucleus pulposus region. Pain and disability were significantly reduced at 3, 6, and 12 months after injection [34]. While disc height restoration was not observed, MRI revealed a substantial increase in water content within the nucleus pulposus after 12 months [34]. Similar outcomes were reported by Pettine et al. with a reduction of one modified Pfirrmann grade at 12 months in eight of 20 treated patients [35]. Improvement in pain and disability was more rapid in patients receiving a higher dose of BMSCs but was reduced in patients older than 40 years, thus indicating that the regenerative efficacy of BMSCs may depend on cell dosage and patient-specific factors [35]. The first randomized controlled trial evaluating the effectiveness of intradiscal stem cell therapy for IDD was conducted by Noriega et al. who allocated 24 patients with degenerative LBP to receive either sham infiltration or allogeneic BMSCs from healthy donors [36]. A significant improvement in pain and disability was observed in the BMSCs group at 3 months, and this was maintained throughout the follow-up period [36]. Despite the absence of significant differences in disc height and water content between the groups, a statistically significant improvement in Pfirrmann scores was observed in the treated discs [36]. Overall, stem cell transplantation has yielded promising results in human clinical trials for the treatment of IDD. A growing body of preclinical research focused on IDD has demonstrated the safety, feasibility, and efficacy of stem cell therapy, thereby establishing the basis for future clinical applications.

The regeneration of IDD through stem cell therapy provides an attractive approach with promising outcomes in both basic research and clinical studies. However, several questions remain unanswered, and future developments are required. One of the prerequisites for natural tissue regeneration is an exhaustive understanding of the biological processes required for tissue regeneration [11, 31]. Although there is increasing knowledge of stem cells and their niche, whether cells for stem cell therapy can acquire the functional attributes characteristic of nucleus pulposus cells and adapt to the avascular niche remains unclear [3, 11, 13]. Although animal models have demonstrated the feasibility and robust regenerative capacity of stem cells within degenerated discs, direct extrapolation to human conditions is impeded by multiple factors, including biomechanical properties, different disc structures, sizes, cellularity, shorter lifespans, and the non-physiological onset of IDD in animal models [3, 13]. Persistent laboratory exploration is imperative for elucidating the regulatory mechanisms intrinsic to disc cells and their unique environments to thereby potentially amplify the effectiveness of stem cell therapies [11, 13]. Second, variations in cell type and source are other issues in stem cell therapy for IDD [7, 8]. BMSCs are widely used in basic and clinical studies and are recognized as favorable candidates for IDD regeneration in clinical trials due to their availability and proliferative capacity [7, 11, 37]. Although the results of animal and clinical investigations are encouraging, the risk of unintended osteophyte differentiation and tumorigenesis remains a serious concern [11, 30, 31]. Compared to other stem cells, resident stem cells such as NPSCs offer superior suitability for IDD regeneration due to their endurance within the harsh intervertebral disc microenvironment. Nevertheless, the limited understanding of these stem cells, absence of specific surface markers, and lack of purification techniques pose significant barriers [38]. Regarding future directions, the efficacy of allogeneic transplantation in revitalizing degenerated discs in comparison to autogenic transplantation warrants examination considering the clinical appeal of allogeneic applications based on their ready availability [37]. Determining if stem cells directly differentiate or induce the differentiation of local cells into authentic disc cells requires a clear definition of the phenotype and molecular hallmarks of disc cells [37]. The resolution of these conundrums is likely to emerge through cutting-edge basic research and rigorously structured clinical trials. Third, critical issues such as optimal treatment timing after pain onset, stage of degeneration for intervention, and dosage of implanted cells require attention [11, 37]. IDD is influenced by multiple factors, including genetics, aging, mechanical stress, smoking, and obesity [7, 11]. Many IDD conditions are a normal consequence of aging, and the pathological and painful conditions that might be suitable for cell therapy have not been well defined [11, 13]. Therefore, an early stage regenerative approach, before extensive structural changes and the complete exhaustion of local stem cell reservoirs, is advisable [13, 29]. However, increased local cell density via stem cell transplantation could escalate metabolic demands, thus potentially precipitating metabolic rivalry with extant viable disc cells [13]. In the worst-case scenario, this could culminate in the death of both the resident and implanted cells [39]. Therefore, prior to intervention, the cell dosage should be calibrated meticulously in relation to the severity of the IDD. Subsequent research should be devoted to optimizing therapeutic efficacy and decoding the biological processes involved. Systematic comparisons are important to determine the optimal cell quantity and scaffold selection. Defining the window for intervention at the most advantageous degenerative stage is crucial for identifying candidates for future human trials [37].

This study has several limitations. First, citation count is used as an indicator of the impact of a study, and this may not be reliable. Older studies possess more time to receive citations. Therefore, influential papers published in recent years may have fewer citations and may not be included in the top list. Second, the number of citations is typically influenced by multiple factors such as self-citation and may not reflect the objective impact of a study. Third, only the WoS database was assessed to identify the most-cited publications. High-impact studies in other sources such as books, websites, and other databases could not be included in this study.

## Conclusion

For the first time, this manuscript provides an analytical study of the 50 highest-impact articles on stem cells in IDD. This provides a top list of the most influential publications in this field. The current study should disseminate beneficial knowledge to researchers and clinicians, expand the understanding of historical works regarding stem cell research in IDD, and guide further research on this topic.

## Data Availability

All data generated or analysed during this study are included in this article.

## Abbreviations

IDD: Intervertebral disc degeneration
WoS: Web of Science
AAS: Altmetric Attention Score
LBP: Low back pain
BMSCs: Bone marrow-derived stem cells
ADSCs: Adipose-derived stem cells
NPSCs: Nucleus pulposus-derived stem cells
CESCs: Cartilage endplate-derived stem cells
AFSCs: Annulus fibrosus-derived stem cells
SDSCs: Synovial-derived stem cells
